# High Glucose Exposure Impairs L-Cell Differentiation in Intestinal Organoids: Molecular Mechanisms and Clinical Implications

**DOI:** 10.3390/ijms22136660

**Published:** 2021-06-22

**Authors:** Agnese Filippello, Stefania Di Mauro, Alessandra Scamporrino, Roberta Malaguarnera, Sebastiano Alfio Torrisi, Gian Marco Leggio, Antonino Di Pino, Roberto Scicali, Francesco Purrello, Salvatore Piro

**Affiliations:** 1 Department of Clinical and Experimental Medicine, Internal Medicine, Garibaldi-Nesima Hospital, University of Catania, Via Palermo, 636, 95122 Catania, Italy; agnese.filippello@gmail.com (A.F.); 8stefaniadimauro6@gmail.com (S.D.M.); alessandraska@hotmail.com (A.S.); antonino.dipino@unict.it (A.D.P.); robertoscicali@gmail.com (R.S.); salvatore.piro@unict.it (S.P.); 2 Faculty of Medicine and Surgery, “Kore” University of Enna, 94100 Enna, Italy; roberta.malaguarnera@unikore.it; 3 Department of Biomedical and Biotechnological Sciences, University of Catania, Via S. Sofia, 64, 95123 Catania, Italy; sebastiano.torrisi@unict.it (S.A.T.); gianmarco.leggio@unict.it (G.M.L.)

**Keywords:** intestinal hormones, differentiation, enteroendocrine cells, glucotoxicity, intestinal organoids

## Abstract

Intestinal organoids are used to analyze the differentiation of enteroendocrine cells (EECs) and to manipulate their density for treating type 2 diabetes. EEC differentiation is a continuous process tightly regulated in the gut by a complex regulatory network. However, the effect of chronic hyperglycemia, in the modulation of regulatory networks controlling identity and differentiation of EECs, has not been analyzed. This study aimed to investigate the effect of glucotoxicity on EEC differentiation in small intestinal organoid platforms. Mouse intestinal organoids were cultured in the presence/absence of high glucose concentrations (35 mM) for 48 h to mimic glucotoxicity. Chronic hyperglycemia impaired the expression of markers related to the differentiation of EEC progenitors (Ngn3) and L-cells (NeuroD1), and it also reduced the expression of Gcg and GLP-1 positive cell number. In addition, the expression of intestinal stem cell markers was reduced in organoids exposed to high glucose concentrations. Our data indicate that glucotoxicity impairs L-cell differentiation, which could be associated with decreased intestinal stem cell proliferative capacity. This study provides the identification of new targets involved in new molecular signaling mechanisms impaired by glucotoxicity that could be a useful tool for the treatment of type 2 diabetes.

## 1. Introduction

Over the last ten years, the study of type 2 diabetes has been extended with novel physiopathological aspects related to the actions of gastrointestinal hormones. Thanks to the description of the effect of incretins in 1986 and their observation in diabetes [1,2], abnormalities of the incretin axis have acquired a pivotal role in the pathogenesis of type 2 diabetes. To date, it has been recognized that glucagon-like peptide-1 (GLP-1) and gastric inhibitory peptide (GIP), produced by the enteroendocrine L- and K-cells, respectively, constitute >90% of all incretin effects. Thus, these two incretins and enteroendocrine cells (EECs) have represented additional novel potential molecular targets for the management of type 2 diabetes. Indeed, in the last decade, GLP-1 analogs/antagonists have been widely used for type 2 diabetes therapy [3], giving great benefit to the control of glucose blood levels and the prevention of chronic complications, including cardiovascular disease. Considering type 2 diabetes physiopathogenesis from a GLP-1-centric point of view, the understanding of L-cell function and the use of this cell type as a therapeutic target could be of outstanding interest. L-cells, as well as almost all of the other EECs, are affected by both exogenous stimuli (deriving from intestinal lumen) and endogenous cues (deriving from the bloodstream). The specific contribution of these two kinds of stimuli could explain the role of L-cells in diabetes physiology and pathophysiology. However, many aspects are still unknown and under debate. In particular, it is important to understand if during the natural history of diabetes, hyperglycemia firstly affects incretin secretion, or whether the impaired incretin secretion firstly influences blood sugar levels. So far, we do not have the correct answer to this question, as it is like the “the egg or the chicken” dilemma. In other words, we do not know which is the factor that induces diabetes first and if the alteration of incretin effects/levels is a cause or a consequence of diabetes. It is important to understand if hyperglycemia impairs L-cell function or, in contrast, deregulation of EEC secretions induces diabetes onset. In light of these considerations, the study of EEC functions could also allow us to understand the specific contribution of endocrine pancreatic and intestinal secretions in diabetes pathogenesis.

EECs are localized within the intestinal crypts and villi and represent about 1% of all the epithelial cells in the gut [4]. EECs are able to produce more than 20 different hormones and are usually classified according to their hormone production into several cell types. It is important to highlight that each cell subtype does not correspond to a specific gut hormone, because gene transcripts and their products are often co-expressed within EECs, suggesting that these cells have multi-hormonal functions. Accordingly, different gut hormones have been found in separate subcellular vesicles, and these storage structures differ considerably between EECs, suggesting that EECs may comprise specific enteroendocrine phenotypes, within which individual cells show a broad hormonal spectrum and plasticity, likely reflecting transcriptional changes during EEC differentiation [5,6,7,8]. EEC differentiation is a continuous process tightly regulated in the gut; EECs and all the other intestinal epithelial cells arise from intestinal stem cells (ISCs) expressing leucine-rich repeats containing G-protein coupled receptor 5 (Lgr5) located in intestinal crypts that give rise to proliferating transit-amplifying cells (TA). Each of these cells can generate absorptive enterocytes and all secretory cell types (EECs, Paneth cells, and goblet cells). During EEC commitment, some secretory progenitor cells start expressing neurogenin 3 (Ngn3). Later, other transcription factors, such as the neurogenic differentiation factor 1 (Neurod1), which is specific to EEC lineage and it is believed to be expressed by precursors of L-cells, lead to the maturation toward specific hormonal cell types [9]. To date, since the increase of L-cell number could represent a strategy to enhance GLP-1 production in type 2 diabetes treatment, L-cell differentiation has been widely investigated. Several studies have been performed in order to identify potential pharmacologic targets able to modulate L-cell differentiation in intestinal organoids [10,11]. Nevertheless, the regulatory networks controlling the identity and the differentiation program of EECs are still under investigation [8,12].

Over the last few years, it has been shown that some environmental factors, such as chronically high plasma concentrations of glucose, led to dysregulation of numerous transcriptional regulators and genes involved in β-cell differentiation impairing the function and the identity of β-cells in type 2 diabetes [13]. In the gut, in vivo and in vitro studies have showed that L-cells could produce hormones that are different from GLP-1 during diabetes [14,15]. In support of this observation, Therodorakis et al. reported that in duodenal biopsy tissue from subjects with type 2 diabetes, L-cells go through this hormonal shift [16].

To date, to the best of our knowledge, the precise molecular mechanisms underlying this hormonal plasticity in the gut and the role of metabolic stress, such as the high glucose concentration typical of diabetes patients, in modulating the expression of regulatory networks controlling identity and differentiation of EECs have not been investigated. Understanding these mechanisms could open novel therapeutic horizons to preserve/restore EECs in diabetes.

This study aimed to test the hypothesis that prolonged hyperglycemia exposure (glucotoxicity) induces ISC dysfunction, resulting in impaired development and consequently altered gastrointestinal endocrine cell number/function. The dysfunction of these cells could lead to impaired gastrointestinal hormone secretion. To test the effect of glucotoxicity, intended as cellular dysfunction before cell death pathway activation, on EEC differentiation, we used small intestinal organoid platforms. Intestinal organoids represent a reliable tool for the study of pathways involved in ISC differentiation into mature cells. In this study, the gene expression profile of TFs (transcription factors) regulating identity and differentiation of intestinal cells as well as of markers of mature EECs were analyzed using intestinal organoids chronically exposed to high glucose concentration. Finally, the cellular localization of differentially expressed gene products was also evaluated.

## 2. Results

### 2.1. Cell Viability of Organoids Treated with High Glucose

In order to evaluate glucose cytotoxicity, we performed the MTT assay in organoids exposed to increasing glucose concentrations (25 and 35 mM) and in control organoids exposed to 17.5 mM of glucose for different periods of time (24, 48, or 72 h).

**Figure 1:**
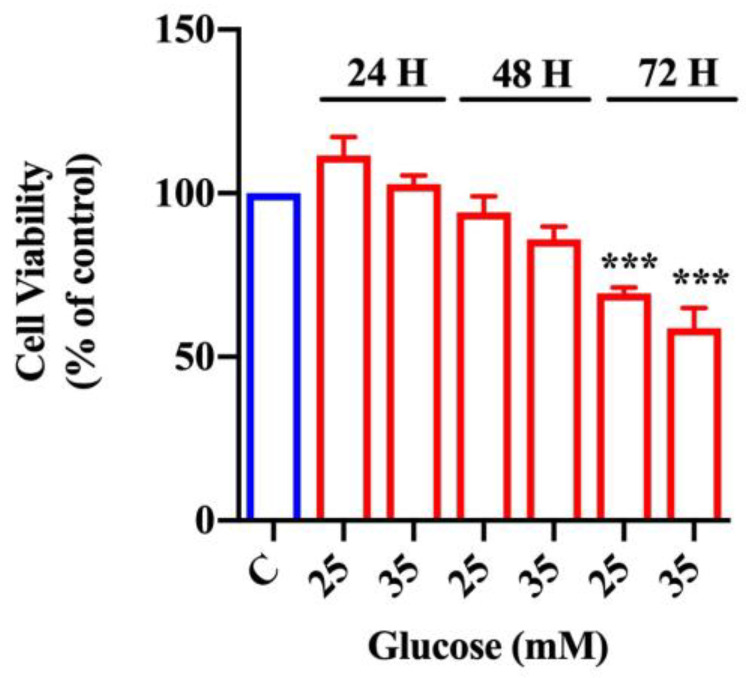
Effect of glucose on cell viability in intestinal organoids. MTT assay in intestinal organoids exposed to increasing concentrations of glucose (25 and 35 mM) after 24, 48, and 72 h (n = 6). Data are expressed as means ± SEM of 570 nM absorbance to % of control. One-way ANOVA followed by Bonferroni test: *** *p* < 0.001 with respect to control (C, 17.5 mM glucose).

As shown in Figure 1, 24 h of glucose treatment did not affect cell viability at all of the tested concentrations. Similarly, exposure to both 25 and 35 mM glucose for 48 h induced a minimal, but not statistically significant, reduction of cell viability with respect to control organoids. On the contrary, glucose treatment for 72 h was toxic compared with control organoids, in a dose-dependent manner and at all the analyzed concentrations. Since glucose treatment for 48 h did not significantly affect cell viability, we chose the highest concentration of glucose (i.e., 35 mM: high glucose). Furthermore, to test the absence of apoptosis, we analyzed the expression of caspase-3 in organoids exposed to 35 mM of glucose for 48 and 72 h; as shown in Figure S1, there were not any significant differences concerning caspase-3 activation compared with control organoids.

### 2.2. Influence of High-Glucose Treatment on Organoid Morphology and Size

**Figure 2:**
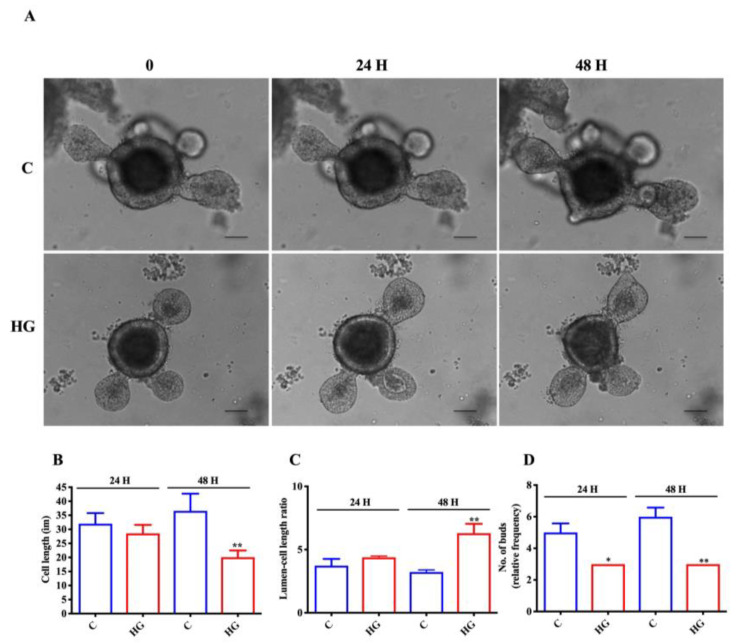
Glucotoxicity affects intestinal organoid growth. (**A**) Representative brightfield images of intestinal organoids following 35 mM glucose exposure for 24 and 48 h. Cell length (**B**), lumen-cell length ratio (**C**), number of buds (**D**) in control, and treated organoids (n = 6). Scale bar, 50 μm. Data are expressed as means ± SEM. Unpaired Student *t*-test: * *p* < 0.05, ** *p* < 0.01. C = Control (17.5 mM glucose). HG = High Glucose (35 mM glucose).

To analyze if high glucose exposure affected organoid growth, we compared the size and some morphological features of intestinal organoids exposed to high glucose concentration (35 mM) at different time points (24 and 48 h) with respect to control organoids (17.5 mM glucose). We found differences between the two types of organoids in their general appearance during growth (Figure 2A). In fact, organoids treated with high glucose for 48 h showed reduced cell length; in contrast, lumen-cell length ratio was increased after 48 h of treatment (Figure 2B,C). We also observed a decreasing trend in the proportion of very budded high-glucose-treated organoids (those with > 3 buds) with respect to control organoids (Figure 2D).

### 2.3. High Glucose Exposure Affects Organoid Stemness Features

**Figure 3:**
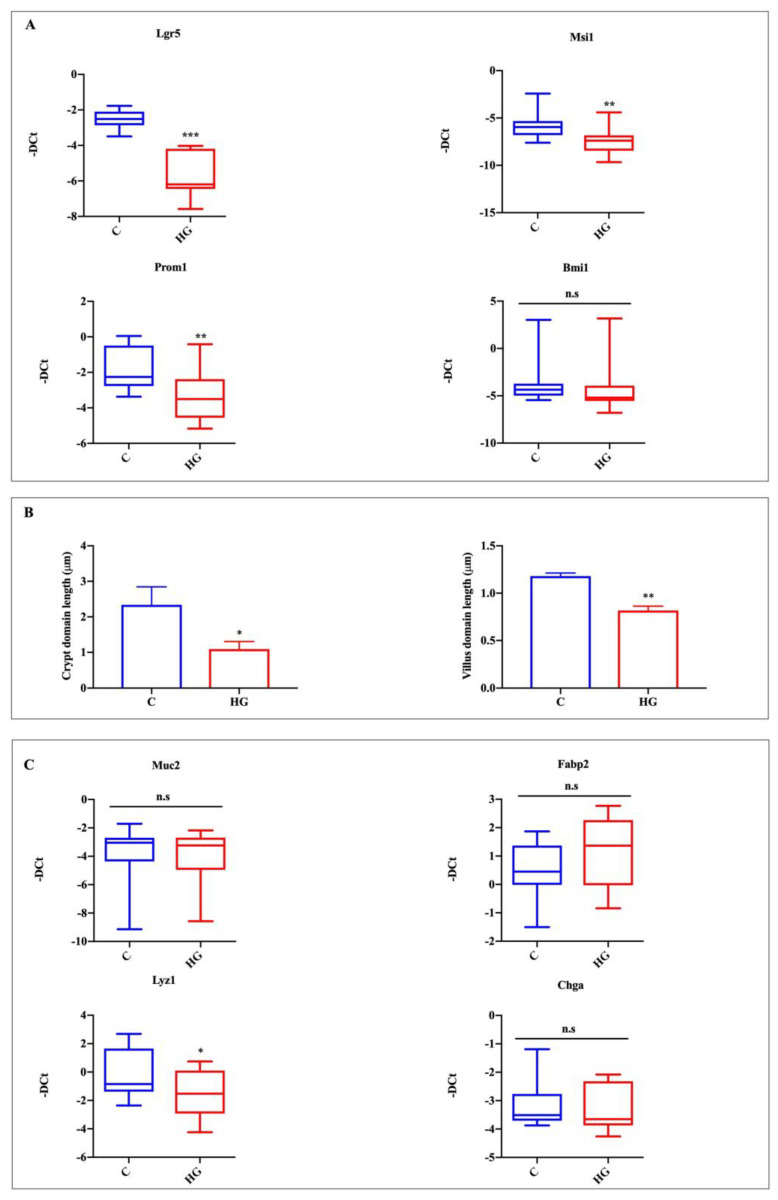
Action of high glucose exposure on intestinal organoid proliferation and specific intestinal epithelial lineage marker expression. Lgr5 and Msi1, proliferating cells, and stem cells; Prom1, TA cells; Bmi1, +4 cells (**A**); crypt domain length and villus domain length (**B**); Muc2, goblet cells; Fabp2, enterocytes; Lyz1, Paneth cells; ChgA, EECs (**C**). Data generated from three biological replicates from five mice. Unpaired Student *t*-test: * *p* < 0.05, ** *p* < 0.01, *** *p* < 0.001; n.s. not significant. C = Control (17.5 mM glucose). HG = High Glucose (35 mM glucose).

In order to test the effect of high glucose exposure on organoid stemness features, we analyzed mRNA expression of stem cell markers such as Lgr5 and musashi RNA-binding protein 1 (Msi1) and the expression of prominin 1 (Prom1) TA cell marker. Finally, we analyzed the expression of the polycomb ring finger oncogene (Bmi1), a marker for reserve intestinal stem cells, which are relatively quiescent and resistant to stress but become activated upon perturbation to active intestinal stem cells [17]. According to growth data, the expression of stem cell and TA cell markers was significantly decreased in organoids exposed to high glucose, while the expression of Bmi1 was unaltered between the two groups (Figure 3A). Moreover, we observed a decreasing trend for the crypt and villus domain in high-glucose-treated organoids compared to controls (Figure 3B). These results suggest a decrease in stem cell proliferative capacity of high-glucose-treated organoids. To evaluate the effect of glucotoxicity on intestinal epithelial lineages, we examined the expression of markers of individual cell types. The expression of the marker for goblet cells, mucin 2 (Muc2), and of the marker for enterocytes, fatty acid binding protein 2 intestinal (Fabp2), were unaltered between controls and high glucose exposed organoids (Figure 3C). The marker of Paneth cells, lysozyme 1 (Lyz1), was significantly decreased in the high glucose treated group, while the EEC marker, chromogranin A (ChgA), was slightly, but not significantly, decreased (Figure 3C).

### 2.4. Effect of Glucotoxicity on the Expression of TFs Associated with Intestinal Cell Differentiation

**Figure 4:**
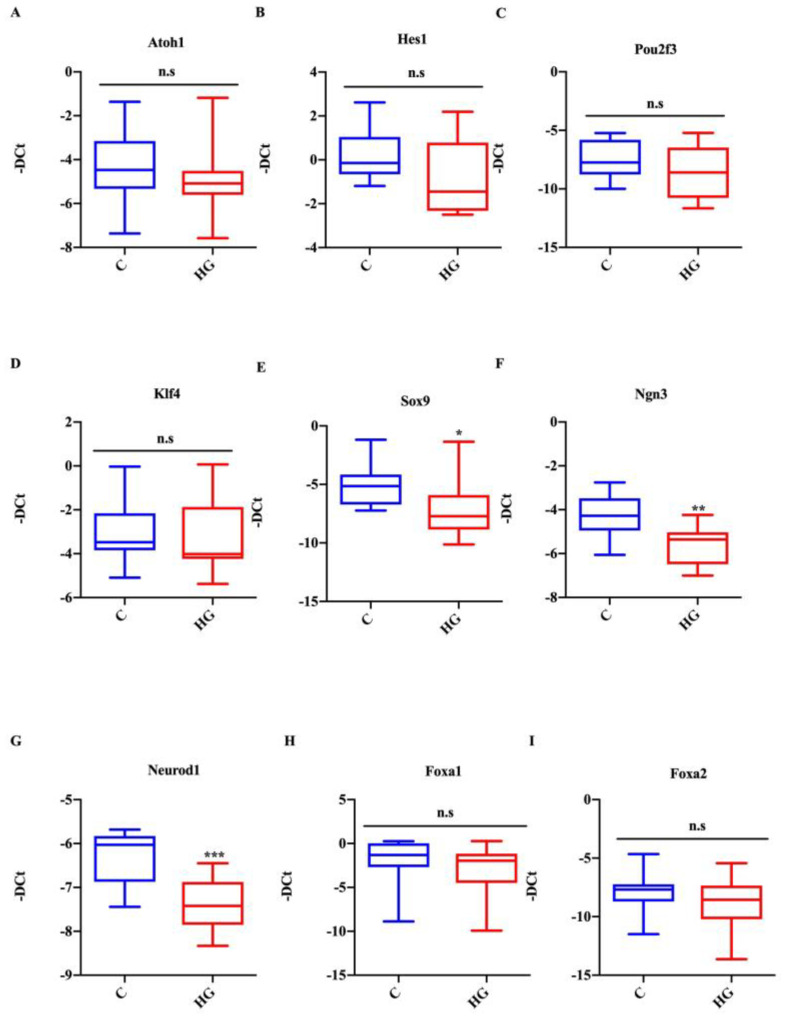
Effect of high glucose treatment on the expression of TFs associated with intestinal cell differentiation. Expression of Atoh1 (**A**), Hes1 (**B**), Pou2f3 (**C**), Klf4 (**D**), Sox9 (**E**), Neurog3 (**F**), Neurod1 (**G**), Foxa1 (**H**), and Foxa2 (**I**) in intestinal organoids cultured with high glucose (35 mM) for 48 h. Data generated from three biological replicates from five mice. Unpaired Student *t*-test: * *p* < 0.05, ** *p* < 0.01, *** *p* < 0.001; n.s. not significant. C = Control (17.5 mM glucose). HG = High Glucose (35 mM glucose).

Mouse small intestinal organoids exposed to high glucose did not show any changes in the expression of atonal bHLH transcription factor 1 (Atoh1) and hes family bHLH transcription factor 1 (Hes1), which are TFs known to be important for secretory and absorptive cell lineages, respectively (Figure 4A,B). Similarly, the Atoh1 and Hes1 expression profile, the expression of the tuft cell marker, POU domain class 2 transcription factor 3 (Pou2f3), and the globet cell marker, Kruppel-like factor 4 (Klf4), were unaffected in controls and the treated group. Conversely, the expression of SRY (sex-determining region Y)-box 9 (Sox9), marker for Paneth cells, was significantly decreased (Figure 4C–E) in the high-glucose-treated group. Interestingly, organoids exposed to high glucose also showed a decreased expression of TFs, which are known to be important for endocrine specification (Figure 4F,G). More in detail, Ngn3, a TF related to the earliest identifiable endocrine progenitor cells, was decreased in the treated group compared with control organoids. In agreement with this result, the expression of NeuroD1, a marker for late endocrine progenitors related to L-cell endocrine specification, was also reduced. In contrast, the expression of forkhead box A1 and forkhead box A2 (Foxa1 and Foxa2), additional markers for L-cell differentiation, remained unchanged (Figure 4H,I).

### 2.5. Glucotoxicity Affects the Identity of EECs

**Figure 5:**
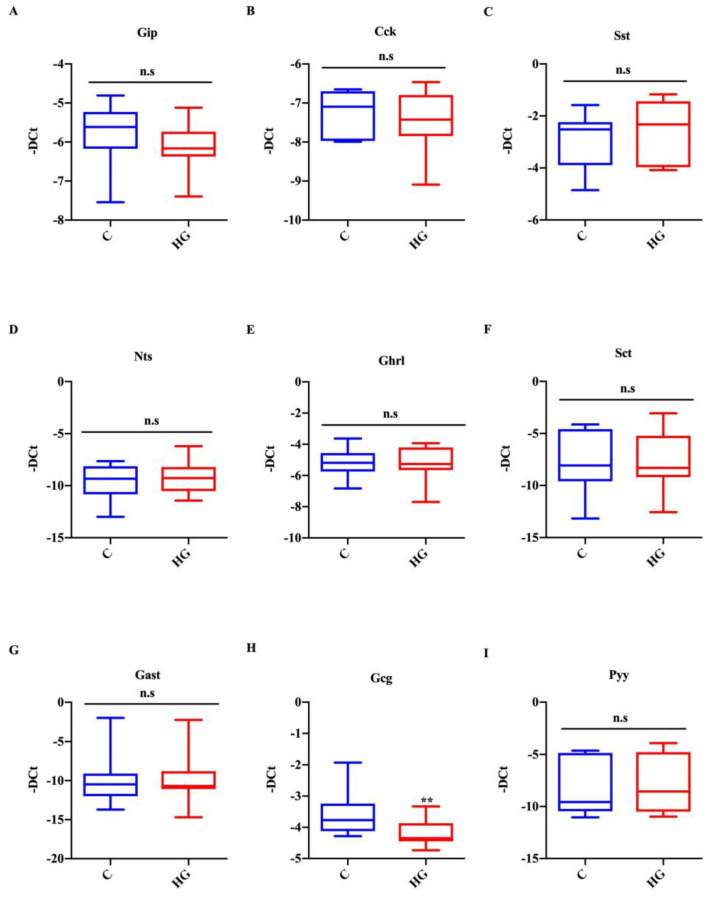
Action of high glucose treatment on EEC markers. Expression of Gip (**A**), Cck (**B**), Sst (**C**), Nts (**D**), Ghrl (**E**), Sct (**F**), Gast (**G**), Gcg (**H**), and Pyy (**I**) in intestinal organoids exposed to high glucose (35 mM) for 48 h. Data generated from three biological replicates from five mice. Unpaired Student *t*-test: ** *p* < 0.01; n.s. not significant. C = Control (17.5 mM glucose). HG = High Glucose (35 mM glucose).

To explore whether high glucose treatment affects the differentiation of early and late endocrine progenitors (positive for Ngn3 and NeuroD1 markers, respectively), we analyzed the expression of EEC markers in organoids exposed to high glucose for 48 h. Using real-time PCR, we observed that the most important gastrointestinal hormones were detectable in intestinal organoids under control conditions. The mRNA expression of markers for K-cells (Gip), I-cells (Cck), D-cells (Sst), N-Cells (Nts), X-cells (Ghrl), S-cells (Sct), and G-cells (Gast) was unchanged in response to glucotoxicity (Figure 5A–G). Similarly to NeuroD1 expression, the mRNA levels of the L-cell marker (Gcg) were significantly decreased (Gcg) in high glucose exposed organoids, while those of another L-cell marker, i.e., Pyy, were unchanged (Figure 5H,I). These results indicate that glucotoxicity may affect L-cell function.

### 2.6. High Glucose Treatment Impairs L-Cell Fate in Intestinal Organoids

**Figure 6:**
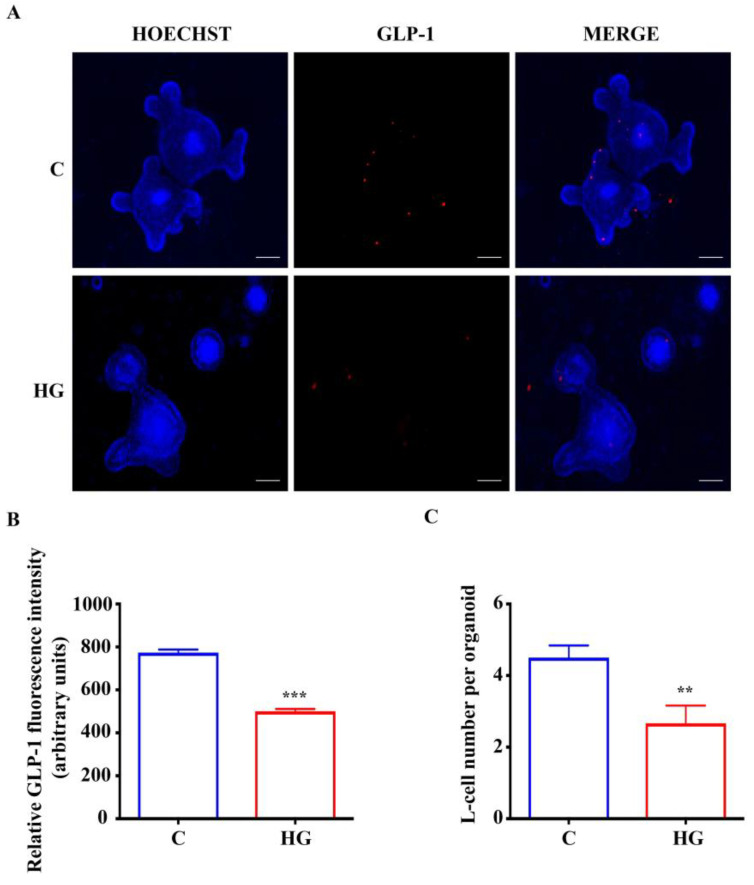
Expression of GLP-1 in small intestinal organoids. (**A**) Representative images of controls and organoids treated with high glucose (35 mM) for 48 h. L-cells, inside organoids, are labeled in red by GLP-1 expression. Nuclei are labeled by Hoechst. Scale bar, 50 μm. (**B**) Quantification of the GLP-1 fluorescence intensity. (**C**) L-cell numbers in control organoids and organoids treated with high glucose. (n = 6). Data are expressed as means ± SEM. Unpaired Student *t*-test: ** *p* < 0.01, *** *p* < 0.001. C = Control (17.5 mM glucose). HG = High Glucose (35 mM glucose).

To investigate if glucotoxicity is able to impair L-cell functional activity, in terms of hormone production, an immunostaining for intracellular GLP-1 was performed in intestinal organoids exposed to high glucose. As shown in Figure 6A,B, the abundance of GLP-1 expression was remarkably decreased in the high glucose organoids with respect to untreated controls. The L-cell number was decreased in high-glucose exposed intestinal organoids compared with controls, consistent with the reduced expression of Gcg and GLP-1 (Figure 6C). These data indicate that glucotoxicity affects the formation of endocrine-committed intestinal cells.

**Figure 7:**
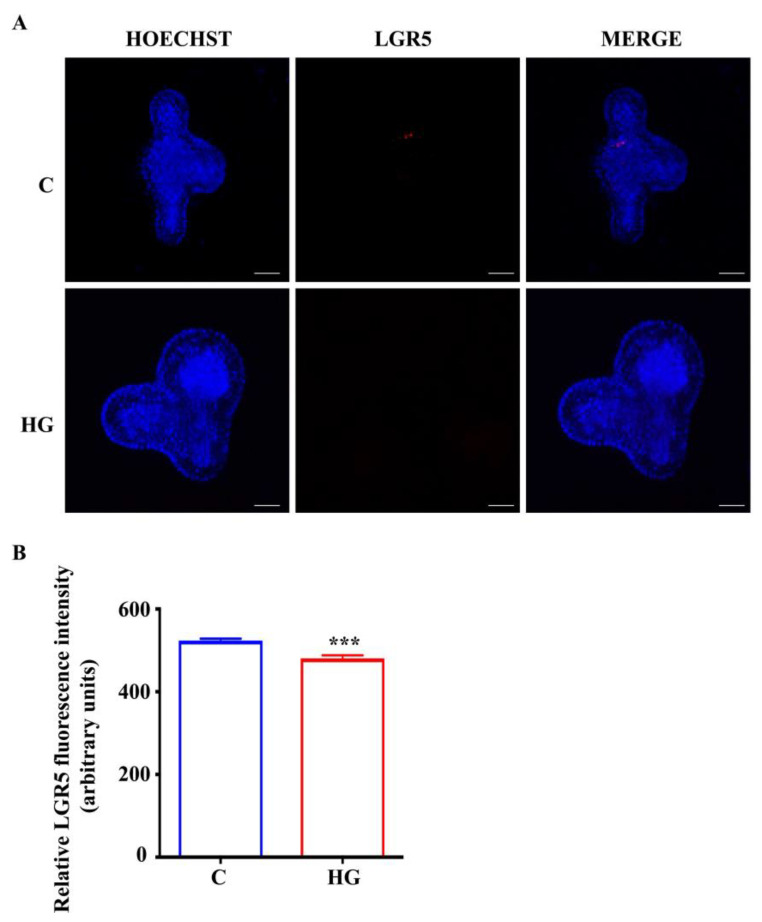
Expression of LGR5 in small intestinal organoids. (**A**) Representative images of controls and organoids treated with high glucose (35 mM) for 48 h. Intestinal stem cells, inside organoids, are labeled in red by LGR5 expression. Nuclei are labeled by Hoechst. Scale bar, 50 μm. (**B**) Quantification of the LGR5 fluorescence intensity. (n = 6). Data are expressed as means ± SEM. Unpaired Student *t*-test: *** *p* < 0.001. C = Control (17.5 mM glucose). HG = High Glucose (35 mM glucose).

According to data about organoid growth and Lgr5 transcript expression, we observed that chronic exposure to high glucose affects organoid development. In order to evaluate if impaired L-cell differentiation in organoids exposed to high glucose is related to reduced Lgr5 gene expression and then organoid growth, we performed the immunostaining for LGR5. The expression of LGR5 was markedly reduced in organoids exposed to high glucose compared with controls (Figure 7A,B).

## 3. Discussion

In our study, we evaluated the effect of chronic hyperglycemia on intestinal cell differentiation using small intestinal organoid platforms that analyzed the involvement of metabolic perturbations in EEC physiology, providing a theoretical basis to the study of diabetes physiopathology. Recent studies have shown that organoids represent a platform to explore the differentiation of intestinal cells and to test the capacity of novel molecules to manipulate the density of EECs for treating metabolic disease [18,19,20]. To date, how diabetes or other metabolic diseases could impair intestinal cell differentiation has not been investigated. For this purpose, by mimicking glucotoxicity in vitro, we analyzed the expression of regulatory networks controlling identity and differentiation of EECs and their functional markers on intestinal organoids.

In this work, we provide evidence that chronic exposure to high glucose (35 mM glucose for 48 h) impairs the expression of TFs, Ngn3, and Neurod1, related to the differentiation of early and late endocrine progenitors, respectively. In particular, the altered NeuroD1 expression, a widely known marker associated with L-cell endocrine specification, in our cell model could lead to impaired L-cell differentiation. In addition, we demonstrate for the first time that altered L-cell differentiation could be associated with decreased stem cell proliferative capacity induced by chronic hyperglycemia.

The expression of TFs regulating the differentiation of EECs has been extensively studied in mouse and human intestinal organoids [8,21]. NGN3 is the most important TF regulating the secretory cell progenitor commitment towards endocrine differentiation; all EECs derive from NGN3-positive cells [22,23]. It has been reported that the absence of NGN3 is associated with impaired EEC development and diabetes onset [22,24]. According to this evidence, we showed that chronic hyperglycemia reduced the expression of Ngn3 transcript in intestinal organoids exposed to high glucose compared to controls. Since Neurod1 is downstream of Ngn3, we also analyzed Neurod1 expression and observed that it was significantly reduced. It is important to note that several studies showed that Neurod1 and Foxa1/2 are TFs specifically associated with L-cell development [9,25]. We further explored the expression of the Ggc L-cell marker and found that, coherently with Neurod1 expression, its expression was significantly decreased in organoids exposed to high glucose compared to controls. Recently, Lund et al. reported that lithocholic acid and L3740 (a synthetic GPBAR1 agonist) increased the L-cell number in intestinal organoids inducing the expression of Gcg, Ngn3, and NeuroD1, while the expression of Foxa1/2 was unchanged [20]. According to this study, we demonstrated that glucotoxicity decreased the Ngn3-Neurod1-Gcg expression pattern, leading to a reduction of L-cell number, as demonstrated by immunofluorescence analysis of GLP-1 positive cells. Several studies have shown that also other intestinal epithelial cells (enterocytes, tuft cells, goblet cells, and Paneth cells) are impaired during metabolic diseases [26,27,28]. To test whether glucotoxicity was able to impair intestinal epithelial cell differentiation, we analyzed the effect of high-glucose treatment on the expression of epithelial cell markers and TFs regulating different intestinal lineage fates in intestinal organoids. In our experimental model, we found that only the expression of Sox9, a key factor of Paneth cell differentiation, was reduced in intestinal organoids exposed to high glucose; in agreement with this data, we found that another marker of Paneth cells, i.e., Lyz1, was reduced in the presence of high glucose. It has been reported that, in endocrine pancreatic cells, Sox9 plays a key role in the regulation of Ngn3 expression, which is essential for the formation of all islet cell types [29]. Since we found that glucotoxicity altered the expression of both Sox9 and Ngn3, we could suppose that also in our in vitro model of intestinal organoids, Ngn3 and Sox9 expression levels are correlated. Similarly to Paneth cells, Lgr5+ cells also play a critical role in intestinal organoids; indeed, these cells are involved in cell metabolism homeostasis implicated in stem cell maintenance and differentiation in the adult population [30]. Lgr5 is one of the most reliable markers of crypt-base columnar cells (CBCs), and a single Lgr5+ cell is able to generate three-dimensional (3D) in vitro organoid cultures comprising all intestinal cell types [31]. Yilmaz et al., analyzing the role of Lgr5 on intestinal crypt homeostasis, found that caloric restriction increases Lgr5+ cells and Paneth cells, improving intestinal cell metabolism; however, the effect of high-carbohydrate foods on intestinal crypt homeostasis still remains unknown [32]. Consequently, we analyzed the expression of Lgr5 in intestinal organoids exposed to high glucose, and we observed that glucotoxicity significantly reduced Lgr5 expression. Therefore glucotoxicity, by reducing LGR5 expression and organoid growth, could affect intestinal crypt homeostasis. Moreover, since it has been reported that a single Lgr5+ cell is able to generate all intestinal cell types, including EECs [31], and the use of molecules increasing L-cell number increases both Ngn3-Neurod1-Gcg pattern and Lgr5 expression [20], we can suppose that the L-cell reduction observed in intestinal organoids under glucotoxicity conditions could be associated with reduced LGR5 expression. In turn, LGR5 reduction could cause an impairment of cell fate and a decrease of stem cell proliferative capacity and consequently a decrease of the EEC lineage. This hypothesis could also be supported by the reduction of organoid length with the contemporary increase of lumen-cell length ratio and by a decreasing trend in the proportion of very budded high-glucose-treated organoids. To demonstrate the potential direct effect of CBCs marked by LGR5 on L-cell differentiation, further studies aimed to analyze the effect of chronic exposure to high glucose on L-cell differentiation in LGR5 positive cells and/or NGN3 positive cells obtained by single cell isolation will be needed. Moreover, to better describe the Ngn3-Neurod1-Gcg pattern involved in L-cell differentiation, global gene expression analysis could be necessary to find if other L-cell differentiation mediators are involved than those we have reported.

The results of this study, in agreement with literature data [33,34,35], add novel elements that can lead to a better understanding of the cell intestinal secretory alterations occurring in diabetes. Although the contribution of gastrointestinal hormones to glycemic homeostasis is known, the pathophysiological bases of these hormone actions are not clear, and whether these gastrointestinal hormone alterations precede or follow diabetes onset is absolutely unknown. Our data demonstrate that chronic hyperglycemia contributes to the deregulation of EEC development and suggest that diabetogenic factors, affecting blood glucose levels, could also impair L-cells and their precursors. Therefore, glucotoxicity could have these novel cellular targets.

A limitation of our study is that we used mouse and not human intestinal organoids. Nevertheless, mouse small intestinal organoids represent an excellent model to study the differentiation of EECs and the interaction among them and other epithelial cell types [36]. Furthermore, it is important to note that the growth of human organoids is more complex than their murine counterparts, and it requires additional activation and inhibition of several pathways. Finally, the differentiation of human intestinal organoids seems to be an endpoint process that exhausts the stem-cell pool and results in a limited lifespan of the resulting cultures of about a week [37].

In conclusion, our data show that chronic exposure to high glucose impairs L-cell differentiation, altering specific TF expression in mouse small intestinal organoids. The impaired L-cell differentiation seems to be also associated with decreased stem cell proliferative capacity induced by glucotoxicity. This study provides the identification of new targets involved in new molecular signaling mechanisms impaired by glucotoxicity that could be a useful tool for better treatments of type 2 diabetes and obesity.

## 4. Materials and Methods

### 4.1. Animals

Intestinal crypts were isolated from male C57BL/6J mice (Charles River Laboratories Italia, Italy, 8–12 weeks old). Animals were group-housed, with free access to chow and water, in an air-conditioned room, with a 12-h light–dark cycle and with constant temperature (23 ± 1 °C) and humidity (57 ± 3%) conditions, as previously reported [38]. All experiments were performed according to EU Directive 2010/63/EU, the Institutional Animal Care and Use Committees of Catania, and the Italian Ministry of Health (authorization n.110/2019 PR).

### 4.2. Crypt Isolation

Intestinal crypts were isolated from the small intestine as previously described [31,39] with minimal adjustments. Briefly, isolated small intestine was opened longitudinally and washed with cold PBS (Sigma-Aldrich, Saint Louis, MO, USA). The tissue was chopped into 5–10 mm pieces that were washed with PBS. Tissue pieces were incubated with ice-cold 30 mM EDTA in PBS for 5 min at 4 °C, transferred to cold PBS, and shaken for a few seconds. This treatment was repeated several times to obtain supernatant fractions enriched with crypts. These fractions were passed through a 70 μm cell strainer (BD Bioscience) to remove residual villous material. Crypts were centrifuged at 200 g for 5 min to separate crypts from single cells.

### 4.3. Organoid Culture

Pellet crypts were resuspended in Matrigel (Corning, New York, USA). Briefly, 50 μL of Matrigel-containing crypts was plated in the center of wells in a 24-well plate. After polymerization at 37 °C, 500 μL of the medium (IntestiCult Organoid Growth Medium (Mouse), STEMCELL Technologies, Vancouver, Canada) was added, and a protocol adapted from manufacturer’s instructions as previously reported was used [31]. The culture medium was changed three times per week. After 7–10 days, organoid cultures were passaged. The organoid medium was removed, and Matrigel including organoids was dissolved with Gentle Cell Dissociation Reagent (STEMCELL Technologies, Vancouver, BC, Canada). Following centrifugation at 200× *g* for 5 min, the supernatant was removed and pelleted organoids were resuspended in Matrigel. All experiments were performed at least after two passages.

### 4.4. MTT Assay

In physiological conditions, mouse intestinal organoids grow in a medium containing 17.5 mM of glucose as previously reported [31,40]; for this reason, we choose this concentration as a control condition. Therefore, before starting the experiment, in order to mimic glucotoxicity metabolic perturbation, intestinal organoids were treated with increasing concentrations of glucose (from 17.5 to 25 and 35 mM) for 24, 48, and 72 h, and cell viability was evaluated using the MTT assay. To create hyperglycemic conditions, the basal medium was modified by adding glucose (Sigma-Aldrich, Saint Louis, MO, USA) at different concentrations (25 mM and 35 mM).

An equal number of organoids was seeded in 96-well plates. After 3–4 days of culture, organoids were exposed to increasing concentrations of glucose (from 17.5 mM to 25 mM and 35 mM) for extended periods (24, 48, and 72 h). After treatment, MTT (Sigma-Aldrich, Saint Louis, MO, USA) assay was performed as previously reported [41,42].

Moreover, to exclude an osmotic effect of the glucose, we performed a set of experiments using mannitol (25 and 35 mM); in these conditions, we did not observe any effect on cell survival.

### 4.5. Evaluation of Mouse Small Intestinal Organoid Growth Treated with High Glucose

Organoid growth and the effect of glucotoxicity on organoid development were analyzed by collecting brightfield images of control (17.5 mM glucose) and high glucose (35 mM glucose) treated organoids, respectively, with inverted fluorescence microscopy TI-E (Nikon, Amsterdam, Netherlands, Europe) using a Mc DS-Qi2 Mono Digital Camera as previously reported [43]. The length of organoids, lumen-cell length ratio, number of buds, and crypt and villus domains were measured using Nis Element AR Software (Nikon).

### 4.6. Total RNA Isolation, Reverse Transcription and Real-Time PCR

After chronic exposure to high glucose concentrations (from 17.5 to 35 mM glucose), organoids were harvested by dissolving Matrigel with cold PBS. Total RNA was extracted from organoids using the RNeasy Mini Kit (Qiagen) according to the manufacturer’s instructions. RNA concentration was determined by spectrophotometric analysis (Nanodrop, Thermo Fisher Scientific, Rodano, MI, Italy). Quantitative real-time PCR was performed using Power SYBR^®^ Green RNA-to-CT™ 1-Step Kit (Thermo Fisher Scientific, Rodano, MI, Italy) as previously reported [44]; we used beta-2-microglobulin (B2M) as the endogenous control gene. Gene expression changes were analyzed with the 2−ΔΔCt method [45].

### 4.7. Immunostaining of Organoids

Immunofluorescence staining of organoids was performed as previously reported by using mouse monoclonal anti-GLP-1 and mouse monoclonal anti-LGR5 primary antibodies (Thermo Fisher Scientific, Rodano, MI, Italy) [18]. Images were acquired on an inverted fluorescence microscope TI-E (Nikon, Amsterdam, The Netherlands, Europe). Image analysis was performed using Nis Element AR Software (Nikon).

### 4.8. Statistical Analysis

Quantitative results are presented as means ± SEM. Comparison of two groups was done using Student’s *t*-test. Comparisons between more than two groups were assessed via ANOVA followed by post hoc analysis for significance (Bonferroni test). A p-value less than 0.05 was considered statistically significant. The statistical analysis was performed using GraphPad Prism 6.0 (GraphPad Software, Inc., San Diego, CA, USA).

## Data Availability

Data are contained within the article or Supplementary Material.

## Supplementary Materials

The following are available online at https://www.mdpi.com/article/10.3390/ijms22136660/s1.
