# Supplementary material for: High Glucose Exposure Impairs L-Cell Differentiation in Intestinal Organoids: Molecular Mechanisms and Clinical Implications
Source: Int J Mol Sci. 2021 Jun 22;22(13):6660. doi: 10.3390/ijms22136660 (PMC8268781; doi:10.3390/ijms22136660)
Supplement: Supplementary file 1 [file ijms-22-06660-s001.zip › ijms-1255577-supplementary.pdf]

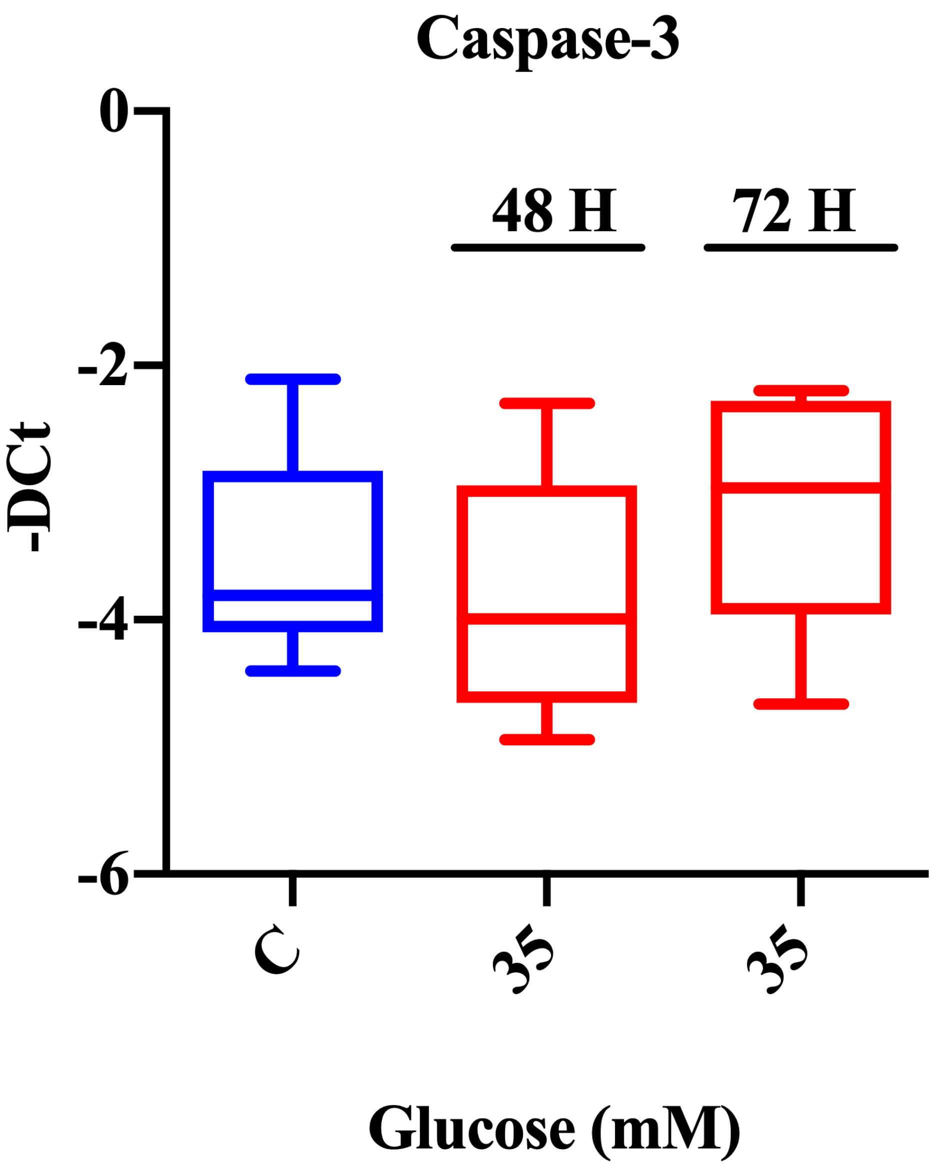

Figure S1. Effect of high glucose on caspase-3 expression.
Expression of caspase-3 in intestinal organoids cultured with high glucose (35 mM) for 48 h and 72 h.
